# Tunneling Nanotube-Mediated Communication: A Mechanism of Intercellular Nucleic Acid Transfer

**DOI:** 10.3390/ijms23105487

**Published:** 2022-05-14

**Authors:** Julia Driscoll, Piyush Gondaliya, Tushar Patel

**Affiliations:** Departments of Transplantation and Cancer Biology, Mayo Clinic, Jacksonville, FL 32224, USA; driscoll.julia@mayo.edu (J.D.); gondaliya.piyushkumar@mayo.edu (P.G.)

**Keywords:** tunneling nanotubes, nucleic acid transfer, intercellular communication

## Abstract

Tunneling nanotubes (TNTs) are thin, F-actin-based membranous protrusions that connect distant cells and can provide e a novel mechanism for intercellular communication. By establishing cytoplasmic continuity between interconnected cells, TNTs enable the bidirectional transfer of nuclear and cytoplasmic cargo, including organelles, nucleic acids, drugs, and pathogenic molecules. TNT-mediated nucleic acid transfer provides a unique opportunity for donor cells to directly alter the genome, transcriptome, and metabolome of recipient cells. TNTs have been reported to transport DNA, mitochondrial DNA, mRNA, viral RNA, and non-coding RNAs, such as miRNA and siRNA. This mechanism of transfer is observed in physiological as well as pathological conditions, and has been implicated in the progression of disease. Herein, we provide a concise overview of TNTs’ structure, mechanisms of biogenesis, and the functional effects of TNT-mediated intercellular transfer of nucleic acid cargo. Furthermore, we highlight the potential translational applications of TNT-mediated nucleic acid transfer in cancer, immunity, and neurological diseases.

## 1. Introduction

The ability of cells to communicate with one another is a prerequisite for multicellular organisms, and is necessary for the physiological functioning of cellular systems, tissues, and organisms. In pathophysiological settings, intercellular communication can contribute to disease onset and progression, as well as to cellular, tissue, or organismal survival in response to adverse environmental stress. Intercellular communication can occur through different mechanisms involving both directed, contact-based communication and non-directed, distant communication, such as through protein or vesicle release [1,2]. Contact-dependent or juxtacrine signaling can be mediated through tunneling nanotubes (TNTs), gap junctions, or the binding of a surface-bound ligand to its cognate receptor [2,3]. TNTs provide directed communication between cells, and comprise membranous connections that enable the exchange of biological cargo between cells [4,5]. Following their description in PC12 pheochromocytoma cells by Rustom et al. [6], TNTs have been reported in many different normal or diseased cells and tissues, and have been implicated in many diverse biological activities [4,5,7,8,9,10].

TNTs are described as tubular extensions between two remote cells composed of filamentous actin (F-actin), with diameters ranging from 50 to 200 nm, and with maximum lengths that can span the distance of several cells [4]. A unique functional feature of TNTs is their ability to transfer different types of cargoes between connected cells [4,10,11]. These include organelles, pathogens, ions, genetic material, and misfolded proteins [12,13,14,15,16,17]. TNTs enable rapid and efficient transfer of these cargoes between cells in a directed manner [18]. Their contribution to functional dynamic multicellular interactions underlies their contribution to cellular and tissue physiology, and subsequent biological roles in physiological and pathological processes [10,19,20]. 

While detailed characterization of the ultrastructure and molecular composition of TNTs is emerging through the use of advanced imaging technology, there are no unique or specific markers for TNTs. Studies of TNTs from different cell types and settings reveal cell-type-specific diversity in structural features such as length, cargo trafficking ability, and mechanisms of biogenesis [6,11,19,21,22]. Consequently, the defining characteristics of TNTs have been arbitrary. At present, TNTs can be identified on the basis of morphological and functional criteria—(1) they are F-actin-based membranous protrusions that connect at least two cells, (2) enabling the bidirectional transfer of a diverse array of cargo, and (3) appear to hover above the substratum [21,23]. 

## 2. Morphology and Structure of TNTs

Morphological and structural criteria are used for the description of TNTs in vitro. TNTs are membranous filaments that connect at least two cells and can provide cytoplasmic continuity. TNT lengths can span several cell diameters, whereas their own diameters range from 50 to 200 nm [4]. The length and diameter of TNTs differ based on the cells of origin and their protein constituents. TNTs are predominantly composed of F-actin, but can also contain other cytoplasmic and cytoskeletal-related proteins, such as tubulin and microtubules. Their wide heterogeneity in diameter can be partly attributed to the presence of these other proteins [15,24,25]. TNTs containing these additional cytoskeletal proteins are commonly referred to as *thick* TNTs, whereas *thin* TNTs describe nanotubes comprised of only F-actin [15]. The non-actin constituents within TNTs may provide structural support to assist with the long-distance transport of larger cargo, such as organelles or vesicles [15]. Lastly, TNTs do not attach to the substratum, thereby rendering them sensitive to mechanical forces. 

In vivo studies describe TNTs as thin, actin-based filamentous structures that connect at least two cells and facilitate the transport of cargo between cells. The TNTs formed in vivo do not need to establish cytoplasmic continuity between cells to transport cargo. In the retina, TNTs were found to have one terminal that was continuous with the cytoplasm, whereas the other terminal was connected to a gap junction [26]. This subtype of TNT, referred to as *closed-end TNTs*, has also been observed in T cells in vitro [22]. 

Ultrastructural features of TNTs detected using cryo-correlative light and electron microscopy were described in detailed studies of murine CAD and human SH-SY5Y neuronal cells. Notably, TNTs were shown not to exist as singular hollow tubes, but rather as structures that contained 2–11 individual TNTs (iTNTs), held together by N-cadherin. Each iTNT comprised an F-actin bundle arranged in a parallel orientation and with an average diameter of 123 nm. The TNTs between neuronal cells established cytoplasmic continuity, enabling bidirectional transfer of cargo between cells. Analysis of cargo trafficking revealed that the vesicular and organelle cargo could be transferred either within a single iTNT or within the luminal space between several iTNT units. Intra-iTNT cargo transport was facilitated by the molecular motor myosin X (Myo10), and appeared to cause a transient bulging-out of the iTNT [27]. 

### 2.1. Mechanisms of TNT Biogenesis and Formation

Several mechanisms have been proposed for TNT formation, and include actin-driven formation and cell dislodgement [6,19,20,28,29]. Actin-driven TNT formation involves an F-actin-driven de novo process. This begins with the formation of a filopodia-like protrusion from the plasma membrane of a donor cell that elongates and eventually fuses with a neighboring cell, resulting in a TNT. Once an intercellular connection is formed, the cells can continue to develop additional TNTs with other neighboring cells, thus engendering a network of interconnected cells [6]. In several studies of TNT-mediated intercellular communication, treatment of cells with actin inhibitors greatly diminished TNT formation and subsequent intercellular cargo transfer [25,30,31]. These findings support the F-actin-driven mechanism of TNT biogenesis. 

Several molecular mediators of de novo actin-driven TNT formation have been described [25,32,33,34]. While the expression of many of the identified mediators was restricted to myeloid-lineage-derived cells, their ectopic expression in other cell types was sufficient to drive TNT formation [25,32]. One such mediator—leukocyte-specific transcript 1 (LST1)—induced the formation of both thin and thick TNTs through a RelA-dependent mechanism. Upon recruitment to the plasma membrane, active RelA (RelA-GTP) mediated the interaction of LST1 with filamin—a protein that has previously been associated with filopodia formation [35]. Filamin and LST1 were co-localized at the sites of nascent TNT protrusions, and were also present in fully formed TNTs [25]. Another myeloid-lineage-specific protein—M-sec—has also been implicated in de novo formation of thin TNTs [32]. Expression of M-sec as well as exocyst complex component Sec3 has been shown to be necessary for TNT-mediated cargo trafficking [32,36,37]. Other molecular mediators identified as playing a role in de novo TNT formation include myosin X (Myo10) and p53 [33,34]. Knockdown of Myo10 or p53 resulted in a reduction in—but not abolition of—TNT formation, which implies a redundancy in the functional activities of the proteins involved in TNT biogenesis [33,38]. The diversity in the molecular mediators responsible for inducing de novo TNT formation reported in these studies suggests that multiple mechanisms of TNT biogenesis may exist within a given cell type [11,21].

Cell dislodgement is a different method of TNT formation that has been observed in vitro [11,15,20]. TNT formation occurs after two cells that were in close contact with one another become dislodged and move apart, leaving a membrane thread that can subsequently mature into an actin-supported TNT structure. Cell dislodgement resulting in TNT formation has been reported in many types of cells, including macrophages, NK cells, T cells, neuronal cells, and cancer cells [6,15,22,39,40,41]. Some studies suggest that the two cells must maintain contact for at least 4 min before dislodging in order for TNTs to be established [22,39].

These two proposed modes of TNT formation are neither mutually exclusive, nor exclude other potential mechanisms of biogenesis [11,21]. Other mechanisms contributing to biogenesis may reflect the stability of tubular structures due to the intrinsic shapes of phospholipid molecules within biological membranes [29,42]. In some cell types, TNT biogenesis is achieved exclusively via cell dislodgement; however, in other cell types, TNTs are established by both actin-driven and cell dislodgement mechanisms [6,40]. Thus, it is likely that the mechanism of TNT biogenesis may be cell-type-dependent and regulated or influenced by intracellular signaling pathways or paracrine signals present in the microenvironment.

### 2.2. Distinguishing TNTs from Other Types of Cell Protrusions

TNTs represent a specific type of cell protrusion. Other types of canonical cell protrusions include filopodia, microvilli, spines, cytonemes, and intercellular bridges [43,44,45,46]. While filopodia serve many cellular functions and microvilli are known to increase the apical surface area for absorption, only the latter three abovementioned cell protrusions play roles in mediating intercellular communication (Table 1) [47,48]. However, spines differ from TNTs in that they are found exclusively on neuronal cells, and do not connect neighboring cells [43]. TNTs resemble a specialized type of filopodia, called cytonemes. Like TNTs, cytonemes are thin, membranous protrusions composed of F-actin. Cytonemes facilitate signal transduction by transferring signaling molecules from a donor cell to a nearby recipient cell [44]. The cytoneme-mediated mechanism of cargo delivery differs from that of TNTs in that the former does not establish cytoplasmic continuity. TNTs also resemble intercellular bridges, which form as a result of incomplete cytokinesis [46]. While the cargo-trafficking ability of intercellular bridges is similar to that of TNTs, intercellular bridges are restricted to forming homotypic interactions between two cells of the same cell type [46].

A distinguishing feature of TNTs is their functional ability to transfer cargo of various types and sizes—including organelles such as mitochondria—and structural continuity across the cytoplasm of adjacent cells [49]. However, the use of actin-driven structural or biogenetic mechanisms is a common feature in the formation of many cell protrusions, and the specific determinants of formation of TNTs versus other cellular protrusions remain enigmatic. In addition, it is unclear as to why some cells form open-end TNTs, while other cells form closed-end TNTs, which lack cytoplasmic continuity between the interconnected cells; however, both subtypes of TNTs enable cargo transfer [22,26,49].

**Table 1:** Overview of canonical communicative cell protrusions.

Type of Cell Protrusion	Mechanism of Cargo Transfer	Identity of Cargo	Functional Effects	References
Spines	N/A	N/A	Induce signal transduction in neuronal cells; establish synaptic plasticity	[43]
Cytonemes	Endocytosis of the receptor–ligand complex by the recipient cell	Ions and signaling ligands	Signal transduction	[44,45,50,51]
Intercellular bridges	Direct transfer by establishing cytoplasmic continuity	Nutrients and organelles	Nutrient and organelle exchange; cell synchronization.	[46]
TNTs	Direct transfer by establishing cytoplasmic continuity	Organelles, nucleic acids, viruses, proteins, lipids, and pathogenic molecules	Bidirectional transfer of biological cargo	[52,53,54,55,56,57,58,59,60]

Abbreviations: TNT, tunneling nanotubes.

### 2.3. Modulation of TNT Formation

The existing research on TNTs in physiological and pathological conditions demonstrates that TNT formation is highly sensitive to extracellular environmental stimuli [61,62]. Indeed, TNT formation is greatly augmented by cells exposed to oxidative stress, neurodegenerative oligomers, inflammation, radiation, or trauma, as well as in cells undergoing apoptosis [63,64,65,66,67,68,69]. In fact, the activation of several signaling pathways has been associated with the stress-induced increase in TNT formation [33,34,62,70]. In addition to enhancing TNT formation, exposure to certain types of environmental stress also accelerates the cargo transfer rates [52]. These findings suggest that the TNTs serve to assist cells in adapting to unfavorable environmental conditions.

## 3. Role of TNTs in Transferring Nucleic Acids

**Figure 1:**
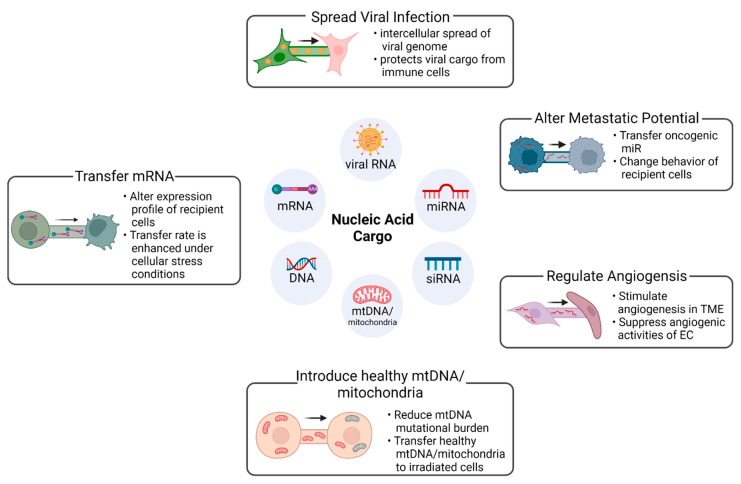
Functional effects of TNT cargo in recipient cells: The TNT-mediated intercellular transfer of nucleic acid cargo has diverse biological effects. This phenomenon is observed in physiological and pathological conditions, and the transfer of cargo can be either bidirectional or unidirectional; the latter mechanism is observed in the spread of several pathological molecules. Abbreviations: EC, endothelial cells; miR, micro RNA; mtDNA, mitochondrial DNA; siRNA, small interfering RNA. TME, tumor microenvironment. This figure was created in BioRender.

TNTs provide an important mode of communication between cells by enabling the bidirectional transfer of intracellular cargo [4,16,18]. This cargo can include organelles, nucleic acids, lipids, pathogenic molecules, and proteins [55,57,58,59,60,71,72]. The transfer of cargo can occur through direct transfer of cytoplasmic constituents, or through facilitated transfer of extracellular vesicles [14]. The exchange of functionally active nucleic acid cargo between interconnected cells by TNTs provides a conduit for a donor cell to genetically modulate gene and protein expression in recipient cells (Figure 1) [72,73]. TNTs have been reported to serve as conduits for DNA and RNA, with intercellular transfer demonstrated for diverse types of nucleic acids, including mRNA, non-coding RNA, viral RNA, and mtDNA (Table 2) [67,72,74,75]. The transfer of nucleic acids through TNTs is distinct from other mechanisms of intercellular transfer—such as through apoptotic bodies or extracellular vesicles—in being both directed and bidirectional, as it involves direct cytoplasmic continuity [74,76,77].

### 3.1. DNA

In a study using laryngeal squamous-cell carcinoma (LSCC) cells, DAPI-stained vesicular-like cargo was observed within the membranous tunneling tubes connecting two LSCC cells. While the identity of the DAPI-stained cargo within the tubular structures was not elucidated, these observations suggest that nuclear DNA could be translocated into the cytoplasm and siphoned between interconnected cells [54]. The possibility that DNA could be directly transferred between cells has important implications for our understanding of the role of genetic influences on cellular physiology within the tissue microenvironment. The directed transfer of DNA between cells via TNTs could modulate cell behavior. In contrast, other modes of transfer of DNA between cells—such as through the release and subsequent uptake of cell-free DNA, or release within EVs—occur in a non-directed manner, and may result in non-specific responses.

### 3.2. Mitochondrial DNA (mtDNA)

The intracellular transfer of mitochondria across cells has long been recognized, and primarily involves TNT-mediated transfer. Mitochondrial transfer between cells has been functionally implicated in cellular responses such as treatment resistance and metabolic plasticity [53,83,84]. Intercellular unidirectional transfer of mitochondria from healthy PC12 neuronal cells to ultraviolet (UV)-irradiated PC12 cells occurred predominantly through TNTs. Mitochondrial DNA (mtDNA) in healthy cells was labeled with ethynyl-2′-deoxyuridine, and was detected within UV-irradiated cells after co-culture. However, it was not determined whether the transferred mtDNA was extramitochondrial mtDNA, or if it was contained within the mitochondria [71]. TNT-dependent unidirectional transfer of mitochondria was also observed between Wharton’s-jelly-derived MSCs (WJMSCs) and patient-derived fibroblasts bearing a point mutation in the mtDNA (mt3243A∆G). Delivery of WT WJMSC-derived mitochondria greatly reduced the mutation burden in the recipient fibroblast cells [78]. The WT mtDNA was detected in the recipient fibroblast cells for up to 28 days in culture. These observations demonstrate that TNT-mediated transfer of mitochondria enables the introduction of donor cell mtDNA to recipient cells.

### 3.3. Messenger RNA (mRNA)

Intercellular transfer of mRNA has been observed between keratinocytes (KCs) and Langerhans cells (LCs), resulting in the delivery of KC-specific mRNA transcripts to LCs. ATAC-sequencing of the recipient cells revealed that the region of chromatin with the KC-specific genes was transcriptionally silent in LCs, confirming that the mRNA transcripts had originated from the KCs. The intercellular transfer of mRNA occurred through a contact-dependent mechanism. Imaging studies revealed networks of TNTs between LCs and KCs, which suggests that TNTs may serve as a conduit for mRNA transfer [73]. Using single-molecule fluorescence in situ hybridization, Haimovich et al. showed that a modified β-actin mRNA transcript bearing a 24-repeat sequence in the 3′ untranslated region could be transferred via TNTs from donor murine embryonic fibroblasts to recipient cells that exclusively expressed WT β-actin. It should be noted that the physiological state of the acceptor cells influenced the rate of mRNA transfer. Following exposure of the acceptor cells to oxidative stress, protein-folding stress, or serum starvation, mRNA transfer rates were increased, whereas heat-shock exposure reduced mRNA uptake by the acceptor cells [52]. Even though the intercellular transfer of mRNA within extracellular vesicles (EVs) is now increasingly recognized, the finite size constraints of EVs imply that only smaller transcripts can be transferred within EVs [75,85]. Thus, TNT-mediated mRNA transfer may provide a more physiological mechanism of intercellular RNA transfer.

### 3.4. Non-Coding RNA

TNTs have been shown to shuttle microRNA (miRNA) cargo between interconnected cells. TNT-mediated miRNA transfer is frequently observed in cancer cells, and can elicit pro-tumorigenic responses in the recipient cells. Several studies have demonstrated the potential of TNTs to transform the local microenvironment to promote tumor growth. In breast cancer, cells with a higher metastatic potential were noted to form more intercellular connections compared with cells with a lower metastatic potential. Nanoscale membrane bridges between MDA-MB-231 metastatic breast cancer cells and endothelial cells (ECs) facilitated the transfer of miR-132—a pro-angiogenic miR—to the ECs. Once delivered to the ECs, miR-132 was functionally active, and modulated the endogenous expression of a downstream target. The observed nanoscale membrane bridges exhibited cytoplasmic continuity between interconnected cells, and were composed of F-actin and tubulin—two of the defining components of TNTs. Pharmacological inhibition of the nanoscale membrane bridges reduced—but did not abolish—miR-132 levels in the ECs. In the absence of TNTs, miR-132 was delivered to the ECs via EVs [14]. TNTs between osteosarcoma (OS) cells and stromal osteoblasts enabled the unidirectional transfer of oncogenic miR-19a to stromal cells. Heterotypic TNTs have also been observed to form between malignant SKOV3 and non-malignant ISOE ovarian cancer cells. These TNTs mediated the unidirectional transfer of miR-199a to ISOE cells. Similarly, high-grade bladder cancer cells transported miR-155 to low-grade bladder cancer cells in a TNT-dependent manner. Upon receipt, the low-grade bladder cancer cells exhibited features characteristic of the high-grade cells [80].

TNT-mediated transfer of miRNA can also support the crosstalk between vascular smooth muscle cells (SMCs) and ECs. Once contact is established between the two cells, TGF-β is secreted by the EC and internalized by the SMC, which subsequently results in the differentiation of the SMC. TNTs formed between differentiated SMCs and ECs enabled the unilateral transfer of the mature miR143/145 cluster to ECs. Upon delivery of the miRNA cluster, the proliferation and angiogenic activities of the recipient ECs were suppressed [56]. TNTs can serve as a conduit for non-coding RNA transcripts other than miRNA. In studies using LSCC cells transfected with fluorophore-labelled double-stranded siRNA, fluorescently labeled cargo was visible within membranous tunneling tubes, and began to accumulate in the recipient cells. Notably, these tunneling tube structures had a closed-end morphology, with one terminal end possessing a gap junction, which served to regulate the entry of the siRNA construct into the recipient cells. [54].

### 3.5. Viral RNA

The formation of TNTs has been shown to be enhanced following viral infection of cells in several reports [55,67,81,82]. TNTs are extremely efficient at spreading viral infections by providing a larger surface for viral entry or propagation, by enabling transfer of viruses from infected to uninfected cells, and by circumventing antiviral defenses or cellular responses. TNTs can facilitate the intercellular transfer of the virus without the death of the host cell. Moreover, TNT-mediated intercellular transfer protects the virus from extracellular antiviral molecules, pre-existing antibodies, immune cells, and/or drugs. Furthermore, TNTs can enable the spread of the entire virion to cells that do not express the cognate receptor(s) required for cell-free viral infection [55]. In addition to the direct spreading of intact virions, TNTs can transfer viral proteins or viral genomes from infected to naïve cells [55,81,82]. The porcine reproductive and respiratory virus (PRRV) was observed to spread through intercellular nanotubes composed of F-actin and myosin IIA. Viral RNA was detected within nanotubes between PRRV-infected MARC-145 cells, and was co-localized with the viral nucleocapsid proteins [55]. However, the functional implications of TNT-mediated viral RNA transfer remain to be established. Vast networks of TNTs have also been observed between PR8-influenza-virus-infected and naïve A549 adenocarcinoma cells. After co-culturing the cells, the PR8 positive-sense genome was detected within the formerly naïve cells. Given the abundance of heterotypic TNTs, the authors speculated that the intercellular viral RNA transfer was mediated by TNTs. However, influenza nucleoprotein expression in recipient cells was unchanged over an 18 h period. Thus, despite the transfer of the viral genome, viral replication may have been impaired by intact intrinsic antiviral activities in recipient cells [81]. Human metapneumovirus (HMPV) viral RNA was also found to spread via a TNT-like mechanism. F-actin-based intercellular extensions that formed between HMPV-infected and naïve lung epithelial cells mediated the unidirectional transfer of viral cargo to the latter cells. Some of the fluorescently labeled viral RNA cargo appeared as large punctate dots, suggesting that structures resembling intact viral nucleocapsids could also be transported via TNTs [82].

## 4. Translational Implications

TNT formation occurs under both physiological and pathological conditions, and is strongly influenced by the state of the local microenvironment.

### 4.1. Cancers

In solid tumors, TNTs may contribute to the maintenance of tissue homeostasis, disruption of which contributes to transformed cell behavior. In tumors, crosstalk by TNTs between tumor cells and stromal cells may enhance tumor growth [86,87]. Within unicellular organisms such as bacteria, the transfer of genetic material provides a mechanism of therapeutic resistance to antibiotics. Demonstration of similar effects with solid tumors will open up new avenues for therapeutic modulation. The potential contributions of TNTs to tumor invasion, metastasis, angiogenesis, metabolic plasticity, chemotherapy resistance, radiosensitivity, bystander effects, and drug delivery have been postulated by several groups [88,89,90].

### 4.2. Immune System

Cells of the innate and adaptive immune systems can participate in TNT-mediated cargo transfer. The role of TNT-mediated nucleic acid transfer in physiological or pathological processes involving immune cells is increasingly being recognized [20,67]. Tunneling nanotubes can contribute to normal physiological functions, such as intercellular antigen trafficking, as well as pathological states, such as the spread of viral or bacterial infections [67,91]. According to several reports, TNTs act in concert with EVs to mediate nucleic acid transfer and intercellular communication [1,14]. While nucleic acid cargo encapsulated within immune-cell-derived EVs can elicit potent immunomodulatory activities on both myeloid and lymphoid cell lineages [92,93], nucleic acid transfer within TNTs could elicit similar responses, but in a direct and targeted fashion.

### 4.3. Neurological Diseases

Although TNTs have been implicated in the spread of pathogenic molecules in several neurological diseases, these cellular conduits represent a unique mechanism for the delivery of therapeutic nucleic acid cargo [12,57]. Since most cerebral parenchymal cells exist in a post-mitotic state, TNT-mediated delivery of RNA-based cargo can be most effective in altering the expression profiles of the recipient cells. Intercellular cargo transfer via TNTs can elicit neuroprotective effects or facilitate the reversion of disease phenotypes by restoring the expression of disease-repressed nucleic acids.

Consequently, understanding the regulation of biogenesis of TNTs and defining their contributions to cell adaptation or survival under adverse conditions may enable targeting these factors to improve therapeutic responses. In settings where TNTs contribute to therapeutic resistance or the spread of pathogens or toxic chemicals, blocking their formation may be desirable. Conversely, in settings where TNTs serve to support cell survival under stress, or facilitate the delivery of drugs or protective factors, supporting their formation may be appropriate. We currently lack knowledge of the mechanisms by which nucleic acids or other cargoes are selected or transported within TNTs, and elucidating these will be necessary to explore the modulation of TNT-mediated transport for translational applications.

## 5. Conclusions

Efficient communication between cells within the tissue microenvironment is greatly facilitated by direct contact provided by TNTs [18]. Intercellular transfer of nucleic acids by TNTs is distinct from other modalities by which this can take place, such as through EVs, direct release, or gap junctions, all of which are very limited, non-directed, and require proximity of cells for optimal effect. This is particularly relevant within highly complex tissue environments such as those within tumors, which may comprise many different types of cells. The functional impact on the recipient cell phenotype and molecular events, as well as the involvement of TNTs in pathophysiological conditions such as viral RNA transmission, highlights the need to further characterize mechanisms of TNT biogenesis and formation, their composition, and the determinants of cargo selection and transfer [74,81]. Targeting these processes may elicit novel approaches for ameliorating disease states in which TNT-mediated nucleic acid transfer occurs.

## Figures and Tables

**Table 2:** Intercellular nucleic acid transfer via TNTs.

Donor Cells	Recipient Cells	Type of Nucleic Acid Cargo	Functional Effects	References
Laryngeal squamous-cell carcinoma (LSCC) cells	LSCC cells	DNA/siRNA	Bidirectional transfer of DNA and siRNA	[54]
Healthy PC12 pheochromocytoma-derived rat cells	Ultraviolet (UV)-irradiated PC12 cells	mtDNA/mitochondria	Unidirectional transfer of mitochondria to UV-irradiated PC12 cells	[71]
WJ-MSC	Patient-derived fibroblasts	mtDNA	Unidirectional transfer of WT mtDNA to patient-derived fibroblasts	[78]
MDA-MB-231 breast cancer cells	Human endothelial cells (EC)	miR-132	Unidirectional transfer of pro-angiogenic miRNA to EC	[74]
K7M2 osteosarcoma cells	MC3T3 murine osteoblast stromal cells	miR-19a	Unidirectional transfer of oncogenic miRNA to stromal cells	[79]
T24 high-grade human urinary bladder cancer cells	RT4 low-grade human urinary bladder cancer	miR-155	Promoted bladder cancer cell reprogramming via activation of the DEPTOR-mTOR pathway	[80]
Primary murine smooth muscle cells	Primary murine endothelial cells	miR-143/145 Cluster	Suppressed the angiogenetic activity of EC	[56]
Murine embryonic fibroblasts (MEF)	Transgenic MBS-MEF	mRNA	Unidirectional transfer of mRNA to WT MEF	[52]
Keratinocytes (KC)	Langerhans cells (LC)	mRNA	Unidirectional transfer of mRNA from KC to LC	[73]
PR8-influenza-virus-transfected A549 human alveolar lung epithelial cells	Uninfected A549 cells	Viral RNA	Spread of viral RNA to uninfected A549 cells	[81]
PRRV-infected MARC-145 monkey kidney cells	MARC-145 cells	Viral RNA	Spread of viral RNA to uninfected MARC-145 cells	[55]
HMPV-infected BEAS-2b human lung epithelial cells	HMPV-infected BEAS-2B human lung epithelial cells	Viral RNA	Spread of viral RNA to near BEAS-2B cells.	[82]

Abbreviations: EC, endothelial cells; HMPV, human metapneumovirus; KC, keratinocytes; LC, Langerhans cells; LSCC, laryngeal squamous-cell carcinoma; MBS, MS2 coat protein (MCP)-binding sequence; MEF, murine embryonic fibroblasts; miR, micro RNA; mtDNA, mitochondrial DNA; PRRV, porcine respiratory and reproductive syndrome virus; siRNA, small interfering RNA.
